# Microstructural and neurochemical plasticity mechanisms interact to enhance human perceptual decision-making

**DOI:** 10.1371/journal.pbio.3002029

**Published:** 2023-03-10

**Authors:** Joseph J. Ziminski, Polytimi Frangou, Vasilis M. Karlaftis, Uzay Emir, Zoe Kourtzi

**Affiliations:** 1 Department of Psychology, University of Cambridge, Cambridge, United Kingdom; 2 Purdue University School of Health Sciences, West Lafayette, Indiana, United States of America; University Medical Center Hamburg-Eppendorf: Universitatsklinikum Hamburg-Eppendorf, GERMANY

## Abstract

Experience and training are known to boost our skills and mold the brain’s organization and function. Yet, structural plasticity and functional neurotransmission are typically studied at different scales (large-scale networks, local circuits), limiting our understanding of the adaptive interactions that support learning of complex cognitive skills in the adult brain. Here, we employ multimodal brain imaging to investigate the link between microstructural (myelination) and neurochemical (GABAergic) plasticity for decision-making. We test (in males, due to potential confounding menstrual cycle effects on GABA measurements in females) for changes in MRI-measured myelin, GABA, and functional connectivity before versus after training on a perceptual decision task that involves identifying targets in clutter. We demonstrate that training alters subcortical (pulvinar, hippocampus) myelination and its functional connectivity to visual cortex and relates to decreased visual cortex GABAergic inhibition. Modeling interactions between MRI measures of myelin, GABA, and functional connectivity indicates that pulvinar myelin plasticity interacts—through thalamocortical connectivity—with GABAergic inhibition in visual cortex to support learning. Our findings propose a dynamic interplay of adaptive microstructural and neurochemical plasticity in subcortico-cortical circuits that supports learning for optimized decision-making in the adult human brain.

## Abstract 2

This multimodal brain imaging study reveals an interactive network of plasticity mechanisms: subcortical microstructural (myelination) plasticity and cortical neurochemical (GABAergic inhibition) plasticity interact through thalamocortical connectivity to support optimized perceptual decisions.

## Introduction

Learning from experience and adapting to changes in our environments is key for skillful actions. Experience and training are known to boost our skills by altering the brain’s structural organization and functional activity. Recent work has challenged the traditional view that structural plasticity is confined to development. In particular, training [1] and neural [2] or sensory [3] stimulation have been shown to promote myelination in the adult brain, the process of insulating neural axons to enhance neurotransmission (for reviews, see [4–6]). Further, training has been shown to alter neurochemical (i.e., GABAergic) signaling that is known to regulate neural activity (for review, see [7]). Yet, microstructural (i.e., myelination) and neurochemical plasticity have mostly been studied at different scales (large-scale networks, local circuits), limiting our understanding of the interactive mechanisms that underlie learning of complex cognitive skills.

Previous studies demonstrate that interactions between myelination, neurochemistry, and activity shape neuronal processing. In particular, myelination of GABAergic interneurons accounts for up to half of the myelin content in the neocortex and disruption of fast-spiking GABAergic interneuron myelination in sensory cortex results in profound deficits in interneuron function [8,9]. Further, both GABAergic interneurons and myelination are thought to promote network synchrony and regulate thalamocortical network oscillations [10,11]. In particular, driving excitatory signals (e.g., visual signals) to GABAergic interneurons promotes inhibitory transmission as a regulator of cortical connectivity [12,13]. Further, feedforward inhibition in sensory cortex has been shown to promote pyramidal output firing to synchronize with gamma-band oscillations associated with intraregional connectivity [12,14]. Yet, how these interactions between myelination, GABAergic inhibition, and functional connectivity shape learning in the human brain remains unknown. In light of previous work in animal models, we hypothesize that interactions between network myelination and visual cortex GABA may shape network connectivity to facilitate learning and plasticity in the human brain.

We employ multimodal brain imaging to investigate the links between microstructural (i.e., myelination) and functional (i.e., neurochemical) mechanisms that regulate sensory processing and support perceptual decisions. We use (a) quantitative MRI to measure myelination markers (i.e., magnetization transfer (MT) saturation) reflecting myelin formation or remodeling; (b) magnetic resonance spectroscopy (MRS) to measure inhibition; and (c) resting-state fMRI (rs-fMRI) to measure functional connectivity. We test whether learning-dependent changes in these MRI-derived markers of brain plasticity predict learning, i.e., our ability to improve after training on a perceptual decision task that involves identifying targets embedded in cluttered scenes [15]. Our results demonstrate a key role of thalamocortical structural (i.e., myelination) and neurochemical interactions for improved perceptual decisions in the adult human brain. In particular, we show that learning to identify targets in clutter alters subcortical (pulvinar, hippocampus) myelination and its functional connectivity to visual cortex and relates to decreased visual cortex GABAergic inhibition. Modeling interactions between these processes suggests that adaptive myelination in pulvinar supports learning for perceptual decisions through thalamocortical interactions with GABAergic plasticity in visual cortex. These adaptive thalamocortical interactions may facilitate selecting task-relevant features from noise early in the training, while visual-hippocampal interactions may refine feature processing for target identification later in the training. Our findings demonstrate a tight interplay between microstructural plasticity and functional neurotransmission mechanisms in subcortico-cortical circuits that interact to support optimized perceptual decisions.

## Results

### Training improves target detection in clutter

**Fig 1:**
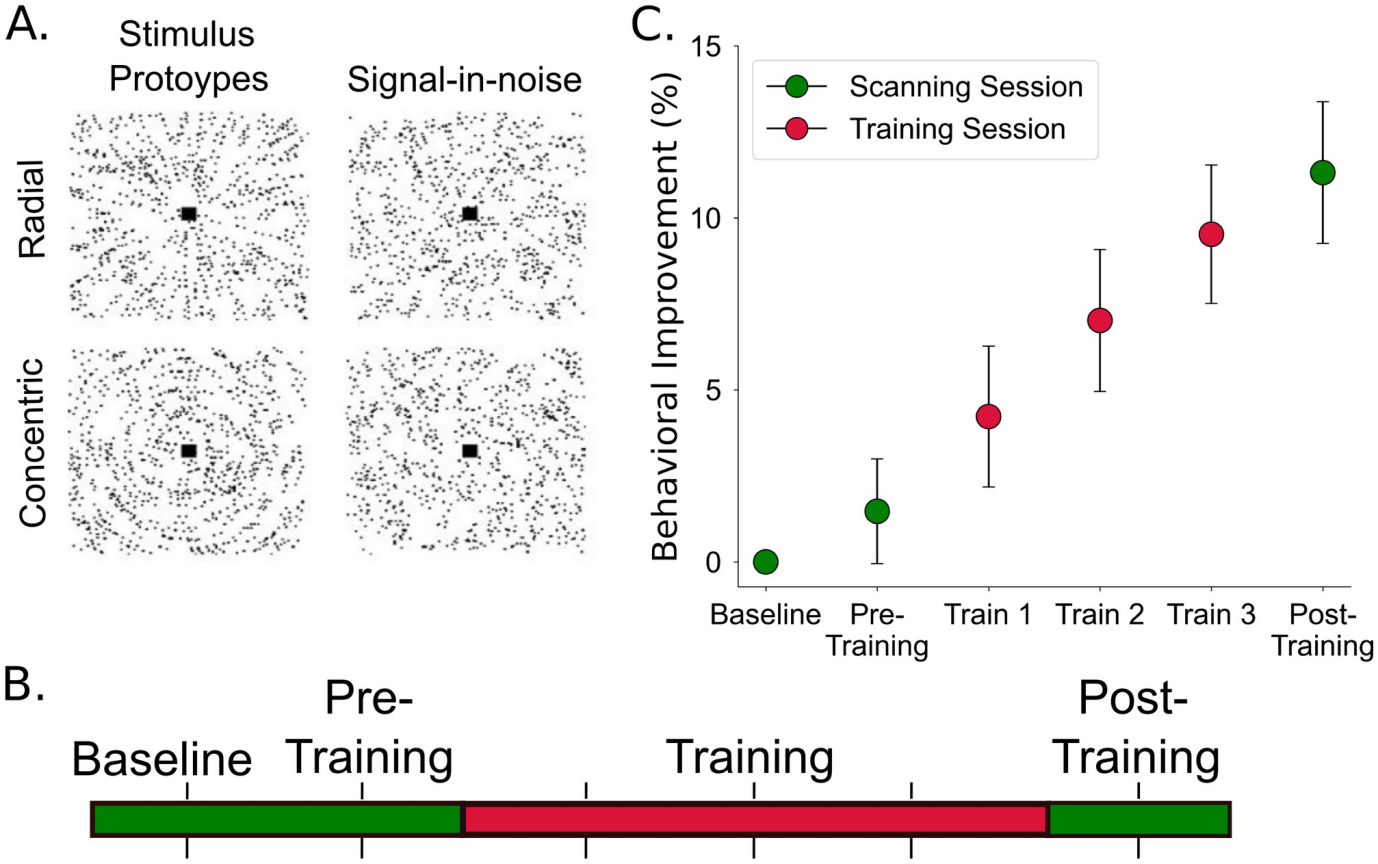
Experimental design and stimuli. **(A**) Radial and concentric Glass patterns are shown with inverted contrast for illustration purposes. Left: Prototype stimuli: 100% signal, spiral angle 0° for radial and 90° for concentric. Right: Stimuli used in the study: 25% signal, spiral angle 0° for radial and 90° for concentric. (**B**) Participants were trained on a signal-in-noise detection task with feedback for 3 consecutive training sessions (one per day). Participants completed the task without feedback in MRI test sessions before (baseline, pre-training) and after (post-training) training. (**C**) Percent behavioral improvement (mean performance per-session minus performance at baseline, divided by performance at baseline) across participants for test (green, in MRI scanner) and training (red, in laboratory) sessions (*n* = 20). Error bars indicate SEM across participants. Source data are provided at: https://doi.org/10.17863/CAM.93457.

We trained participants on a perceptual decision task that involves identifying radial versus concentric dot patterns (Glass patterns) embedded in noise (signal-in-noise (SN) task; Fig 1A). Participants completed 3 behavioral training sessions with feedback and 3 test sessions (during MRI scanning) without feedback (baseline, pre-training, post-training) (Fig 1B). For each participant, we tested for learning-dependent changes in task performance and MRI-derived markers of plasticity before (pre-training) versus after (post-training) training (3 sessions on consecutive days). To test learning specificity, we compared the pre-training session to a baseline session (3 days before the pre-training session) that served as a within-subject no-training control.

Training improved participant performance in the task (Fig 1C; one-way repeated measures ANOVA across sessions, Greenhouse–Geisser corrected; F_5, 95_ = 19.74, *p* < 0.001). In particular, performance accuracy increased in the post-training compared to the pre-training session (Post hoc comparisons, Sidak corrected, *p* < 0.001). This behavioral improvement was specific to training; i.e., there was no significant differences in performance between baseline and pre-training sessions (*p* = 0.997).

### Training alters microstructural myelin plasticity

**Fig 2:**
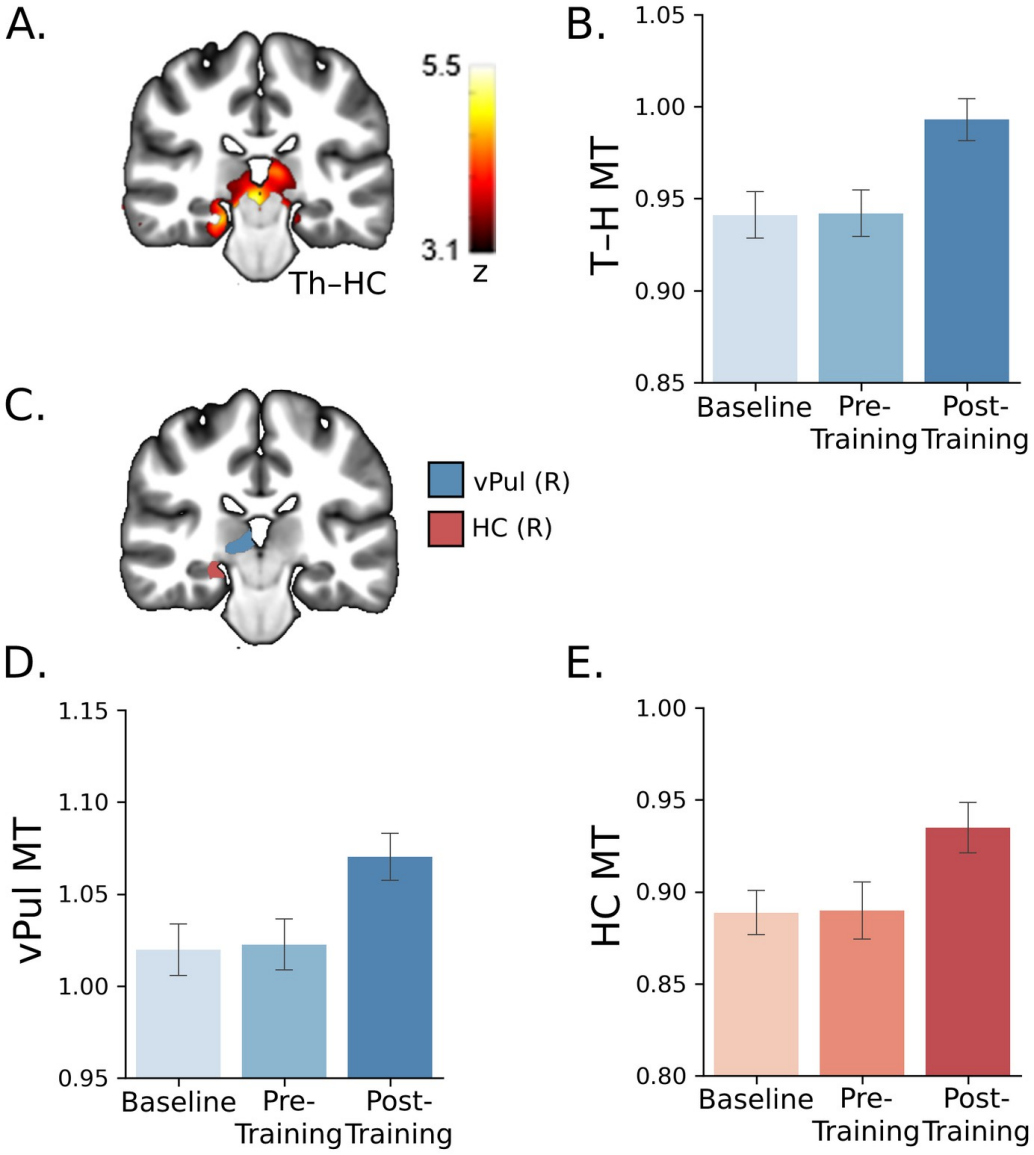
Subcortical MT saturation increases after training and correlates with behavioral improvement. (**A**) Th–HC cluster in MNI space (radiological convention, R-L; vPul x: 9.60 y: −25.60 z: 0.80; HC x: 21.60 y: −27.20 z: −11.20 (mm)) showing significantly higher MT after (post-training) compared to before (baseline, pre-training) training (*p* < 0.001) (S1 Table). (**B)** Mean MT (percentage change from baseline session) in the Th–HC cluster before vs. after training. (**C**) Parcellation of the Th–HC cluster (see Methods for details): vPul (no overlap with LGN), HC (S2 Table). Higher mean MT in (**D**) vPul and (**E**) HC after compared to before training. (*n* = 17). Error bars indicate SEM. Source data are provided at: https://doi.org/10.17863/CAM.93457. MT, magnetization transfer; HC, hippocampus; Th–HC, thalamic-hippocampal; vPul, ventral pulvinar.

To investigate whether training in the SN task alters myelination processes, we tested changes before versus after training (whole-brain GLM) in MT saturation, an MRI indicator of myelin content that has been shown to be measured reliably by multiparameter mapping (MPM) [16–18]. We found significant MT increase in grey matter after training in thalamic-hippocampal (Th–HC), inferior frontal gyrus (IFG), and inferior temporal cortex (ITC) regions (whole-brain repeated-measures GLM; S1 Table and Fig 2A and 2B).

These changes in MT were specific to training; i.e., comparing MT in 2 sessions before training (i.e., whole-brain GLM: baseline versus pre-training) did not show any significant clusters. This lack of significant MT differences between baseline and pre-training sessions suggests the changes we observed in MT following training—within the same participants—were specific to training rather than potential confounds (e.g., pH of the targeted tissue) [19]. Further, these learning-dependent changes were specific to MT—rather than other MRI-derived myelination markers—which have been suggested to reflect changes in myelin formation or remodeling. Vasculature-related signals (i.e., T1 or T2 relaxation rates) may contribute to changes in MRI-derived markers of myelination (MT, R2*, R1). However, we did not find any clusters (whole-brain GLM) that showed significant changes in transverse relaxation rate (R2*), nor any significant (all *p* > 0.05) differences in R2*, R1 across sessions within the clusters that showed MT changes, suggesting that the MT-specific changes we observed could not be simply due to vasculature-related signals.

To provide finer scale analysis of the subcortical cluster, we parcellated the Th–HC region into subregions, using detailed subcortical atlases (Fig 2C–2E and S2 Table). We found that MT change was significantly negatively correlated with behavioral improvement in the ventral pulvinar (vPul; r = −0.49, *p* = 0.045, CI [−0.82, −0.01]) and hippocampus (HC; r = −0.59, *p* = 0.012, CI [−0.90, −0.05]) (S1A and S1B Fig). To ensure that this relationship was not due to variability between participants before training (i.e., at baseline), we regressed out baseline measures and found that the relationships between MT and behavioral change (post-training minus pre-training) remained significant (S1C and S1D Fig: vPul r = −0.58, *p* = 0.015, CI [−0.80, −0.22]; HC: r = −0.62, *p* = 0.008, CI [−0.89, −0.11]). Further analyses on white matter showed significant increase in MT in a white matter cluster adjacent to the grey matter regions (S2A Fig) that related to behavioral improvement (S2B Fig; r = −0.59, *p* = 0.013, CI [−0.81, −0.19]). Taken together, these analyses show that training results in behaviorally relevant changes in MRI markers of myelin (MT) in subcortical regions.

### Training alters functional connectivity in subcortico-cortical networks

Next, we asked whether the learning-dependent changes we observed in myelination relate to changes in functional connectivity, given the role of myelination in enhancing neurotransmission [4–6]. Specifically, we tested whether training alters subcortico-cortical functional networks seeded from the subcortical regions (vPul, HC) that showed myelin plasticity; i.e., we focused on 2 networks: (a) a thalamocortical network including vPul, occipito-temporal cortex (OCT), and anterior cingulate cortex (ACC), which are known to be connected to the pulvinar [20,21] and involved in learning for perceptual decisions [22,23]; and (b) a visual–hippocampal network including HC and visual areas (V1, V2, V3, and V4) that are known to be connected to HC and have been implicated in learning for perceptual decisions [24,25]. This connectivity analysis—despite limitations in capturing whole-brain networks—allows us to target the link between myelin and functional plasticity.

**Fig 3:**
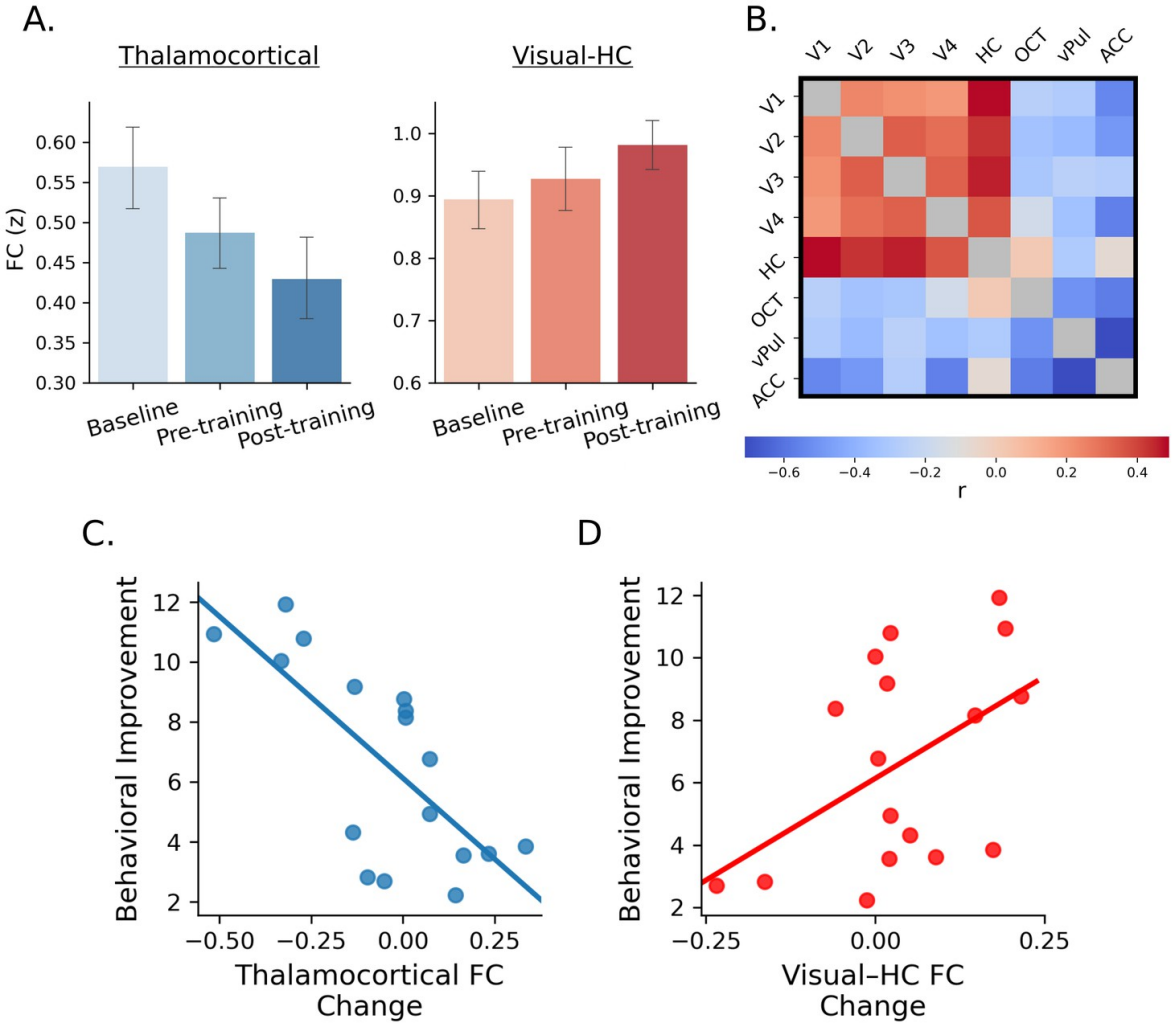
Thalamocortical and visual-hippocampal networks. (**A**) Visual-hippocampal network functional connectivity (V1, V2, V3, V4, HC) increased, while thalamocortical network functional connectivity (OCT, vPul, ACC) decreased after training. (**B**) Correlation matrix (units: Pearson’s r) showing the relationship between change (post-training–pre-training) in functional connectivity between cortical and subcortical regions and behavioral improvement. (**C**) Significant negative correlation between change in mean thalamocortical network functional connectivity and behavior improvement (r = −0.71, *p* = 0.001, CI [−0.88, −0.45]). (**D**) Significant positive correction between change in mean visual-hippocampal network functional connectivity and behavior improvement (r = 0.48, *p* = 0.050, CI [0.03, 0.78]). (*n* = 17). Source data are provided at: https://doi.org/10.17863/CAM.93457. ACC, anterior cingulate cortex; OCT, occipito-temporal cortex; vPul, ventral pulvinar.

We used rs-fMRI to investigate learning-dependent changes in functional connectivity in these subcortico-cortical networks. Previous work has demonstrated a strong link between functional resting-state networks and task-activated networks that have been shown to overlap [26,27]. Reactivation of task-activated networks has been shown to occur at rest following task performance [28,29] and may play a role in memory consolidation [29,30]. Further, previous studies have demonstrated changes in rs-fMRI network activity following training on perceptual or motor tasks [31–35]. First, we found that thalamocortical network connectivity was stronger before training, while visual-hippocampal network connectivity was stronger after training (Fig 3A). In particular, network functional connectivity (i.e., mean connectivity of all network node pairs) across sessions differed significantly between networks; i.e., thalamocortical connectivity decreased, while visual-hippocampal connectivity increased after training (two-way mixed ANOVA, significant network × session (early-training comprising baseline and pre-training versus post-training) interaction: F2,64 = 3.39, *p* = 0.040).

Second, correlating learning-dependent changes (post-training minus pre-training) in functional connectivity in these networks with behavioral improvement showed positive correlations for the visual-hippocampal network, while negative correlations for the thalamocortical network (Fig 3B–3D); i.e., decreased thalamocortical network connectivity (Fig 3C; r = −0.71, *p* = 0.001, CI [−0.88, −0.45]) while increased visual-hippocampal connectivity after training (Fig 3D; r = 0.48, *p* = 0.050, CI [0.03, 0.78]) related to behavioral improvement. These correlations (i.e., thalamocortical versus visual-hippocampal FC correlation with behavioral improvement) were significantly different from each other (z = −3.94, *p* < 0.001) and remained significant when accounting for variability at baseline (i.e., regressing out functional connectivity at baseline; thalamocortical network connectivity: r = −0.72, *p* = 0.001, CI [−0.90, −0.47]; visual-hippocampal connectivity: r = 0.51, *p* = 0.035, CI [0.06, 0.79]). Further, no significant correlations were observed between connectivity differences before training (i.e., pre-training minus baseline) and behavioral improvement (thalamocortical: r = 0.04, *p* = 0.88, CI = [−0.41, 0.44]; visual-hippocampal: r = −0.27, *p* = 0.302, CI = [−0.67, 0.16]), confirming the relationship was specific to the training period. Finally, we did not observe any significant correlations between behavioral improvement and changes in functional connectivity between (a) IFG and OCT (r = −0.12, *p* = 0.647, CI [−0.58, 0.38]); IFG and V1 (r = 0001, *p* = 0.996, [−0.39, 0.51]) and (b) ITC and OCT (r = −0.40, *p* = 0.111, CI [−0.75, 0.05]); ITC and V1 (r = 0.21, *p* = 0.418, [−0.26, 0.63]), suggesting that the learning-dependent changes we observed were specific to subcortico-cortical connectivity.

Taken together, these results suggest that training alters both microstructural myelination processes and functional connectivity in distinct subcortico-cortical networks to support learning for perceptual decisions. Our results showing higher thalamocortical connectivity before training, but higher visual-hippocampal connectivity after training, suggest that thalamocortical and visual-hippocampal networks may contribute to early versus late learning for perceptual decisions, respectively.

### Learning-dependent GABAergic plasticity

**Fig 4:**
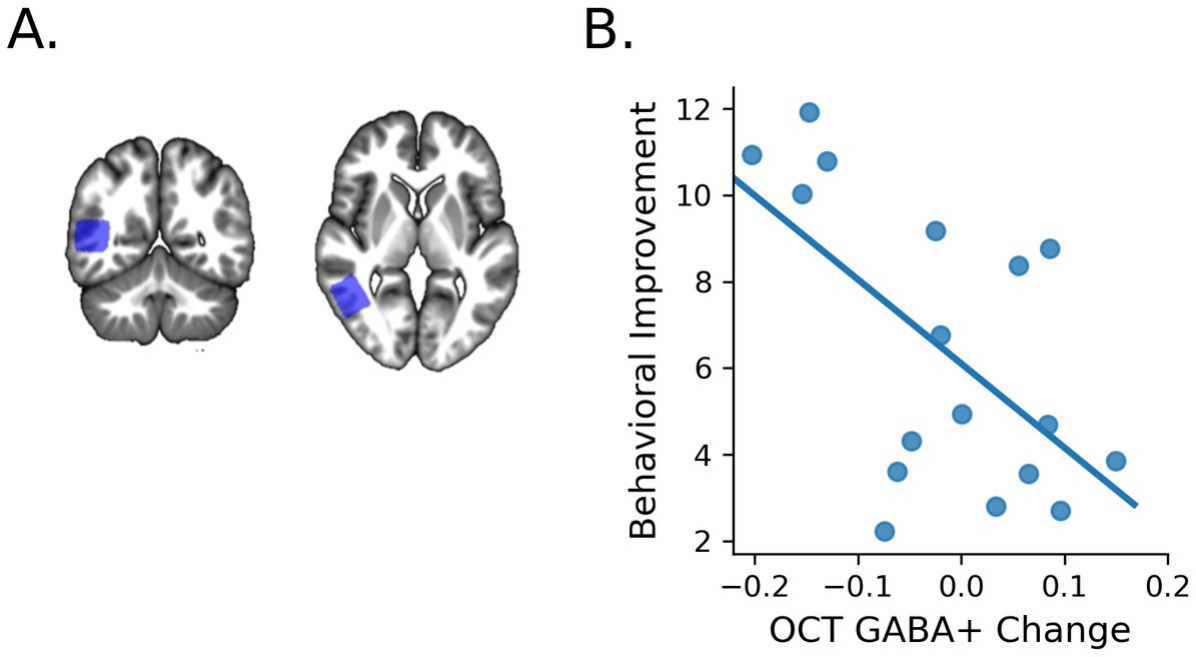
Visual GABAergic plasticity. **(A)** Group MRS voxel mask (cortical region common in 50% or more of participants) indicates OCT voxel placement displayed on the average MT scan across participants (MNI x: 47.2 y: −53.60 z: 8.80 (mm)), in MNI space (radiological convention, R-L). No significant differences in data quality measures were observed across sessions (S3 Table). (**B)** Significant negative correlation between OCT GABA+ change and behavioral improvement (*n* = 17; r = −0.60, *p* = 0.012, CI [−0.85, −0.07]). Source data are provided at: https://doi.org/10.17863/CAM.93457. MT, magnetization transfer; OCT, occipito-temporal cortex.

Previous studies provide evidence that MRS-assessed GABA relates to behavioral improvement due to training, consistent with the role of GABA, the primary inhibitory neurotransmitter, in brain plasticity [15,23,36]. Here, we tested whether GABAergic plasticity in OCT that is known to be involved in perceptual decisions and learning [15,23] relates to behavioral improvement following multisession training (OCT; Fig 4A). In particular, we measured OCT GABA+ during performance on the SN task, before versus after training, to interrogate learning-dependent changes in GABAergic inhibition related to behavioral improvement. We observed a significant relationship between changes in OCT GABA+/water (post-training minus pre-training) and behavioral improvement in the SN task (Fig 4B; GABA+: r = −0.60, *p* = 0.012, CI [−0.85, −0.07]), consistent with the role of decreased GABAergic inhibition in learning-dependent plasticity [7,15,23,37].

To control for the possibility that the learning-dependent changes we observed in OCT GABA+ were due to differences in task difficulty across sessions (i.e., the task was more difficult before than after training), we analyzed reaction times as a measure of task difficulty. Analysis of reaction times did not show any significant relationship between OCT GABA+ change and reaction times differences before versus after training (r = −0.19, *p* = 0.51, [−0.50, 0.13]). Further, we measured GABA+ in posterior parietal cortex (PPC) that is known to be involved in attentional processing [38,39]. We reasoned that any potential differences in attention due to task difficulty would result in differences in PPC GABA+ across sessions. However, we did not observe a significant correlation between PPC GABA+ change and behavioral improvement (S3C and S3D Fig; r = 0.36, *p* = 0.154, CI [−0.22, 0.72]; Steigers Z comparison showed that the correlations of GABA+ change in OCT versus PPC with behavioral improvement were significantly different z = −3.16, *p* = 0.002). This is consistent with our previous work [15] showing dissociable task-dependent GABAergic plasticity in OCT and PPC; i.e., GABA+ decrease due to training was specific to OCT for the SN task (compared to GABA+ increase for training on a Feature Differences task). Finally, during scanning (pre, post-training session) participants did not receive feedback, suggesting that learning-dependent changes in GABA+ relate to performance that is sustained following training.

Additional analyses (S3 Fig and S3 Table) tested the specificity of our results. First, the relationship between GABAergic plasticity and improved perceptual decisions was specific to training; i.e., (a) the relationship between OCT GABA+ change and behavioral improvement remained significant following regression of baseline measures (S3A Fig; r = −0.65, *p* = 0.005, CI [−0.89, −0.25]; and (b) there was no significant correlation between differences in GABA+ and behavior for sessions before training (i.e., pre-training minus baseline (r = −0.03, *p* = 0.917, CI [−0.47, 0.39]). Second, this relationship remained significant when controlling for voxel tissue composition (alpha correction of grey matter concentration; r = −0.64, *p* = 0.006, CI [−0.87, −0.28]; regression of GABA+ concentration with CSF voxel concentration; r = −0.60, *p* = 0.011, CI [−0.85, −0.17]; 1 –fCSF division of GABA+ concentration; r = −0.60, *p* = 0.010, CI [−0.84, −0.17]) and reference metabolite (i.e., NAA rather than water; r = −0.60, *p* = 0.011, CI [−0.81, −0.33]). Third, the relationship between OCT GABA+ change and behavioral improvement was specific to (a) GABA+ rather than other metabolites (i.e., glutamate; r = 0.23, *p* = 0.375, CI [−0.42, 0.76]; Steiger’s Z comparison showed that the correlations of GABA+ change versus glutamate change with behavioral improvement were significantly different; z = −2.69, *p* = 0.007).

Taken together, our results demonstrate a strong relationship between decrease in visual GABAergic inhibition and learning for improved perceptual decisions. Limitations in MRS brain coverage meant that our study focused on specific areas (i.e., OCT versus PPC), providing evidence for the specific role of visual GABAergic plasticity in learning rather than general task engagement. Future work is needed to investigate GABAergic plasticity across areas in the subcortico-cortical networks we showed to be involved in learning for perceptual decisions.

### Linking microstructural, functional, and neurochemical plasticity to learning

We next asked whether the learning-dependent changes we observed in visual thalamus and its functional connectivity to visual cortex link to GABAergic plasticity to support learning for perceptual decisions.

First, we conducted a mediation analysis to model interactions between microstructural (i.e., myelin), functional (i.e., functional connectivity), and neurochemical (GABAergic inhibition) plasticity. This analysis showed that pulvinar myelin plasticity influences GABAergic processing in visual cortex through thalamocortical connectivity (total effect c = 0.44, z = 2.07, *p* = 0.038, [0.024, 0.85]). In particular, the effect of learning-dependent changes in pulvinar MT (predictor) on OCT GABA+ (outcome) was mediated by changes in thalamocortical network connectivity (mediator): indirect effect: ab = 0.36, z = 1.99, *p* = 0.046, [0.01, 0.71]; no significant direct effect c’ = 0.08, z = 0.39, *p* = 0.696, [0.32, 0.47]. These results suggest that learning-dependent changes in pulvinar myelination drive changes in thalamocortical connectivity that gates sensory processing (i.e., gain control) through GABAergic inhibition in visual cortex.

**Fig 5:**
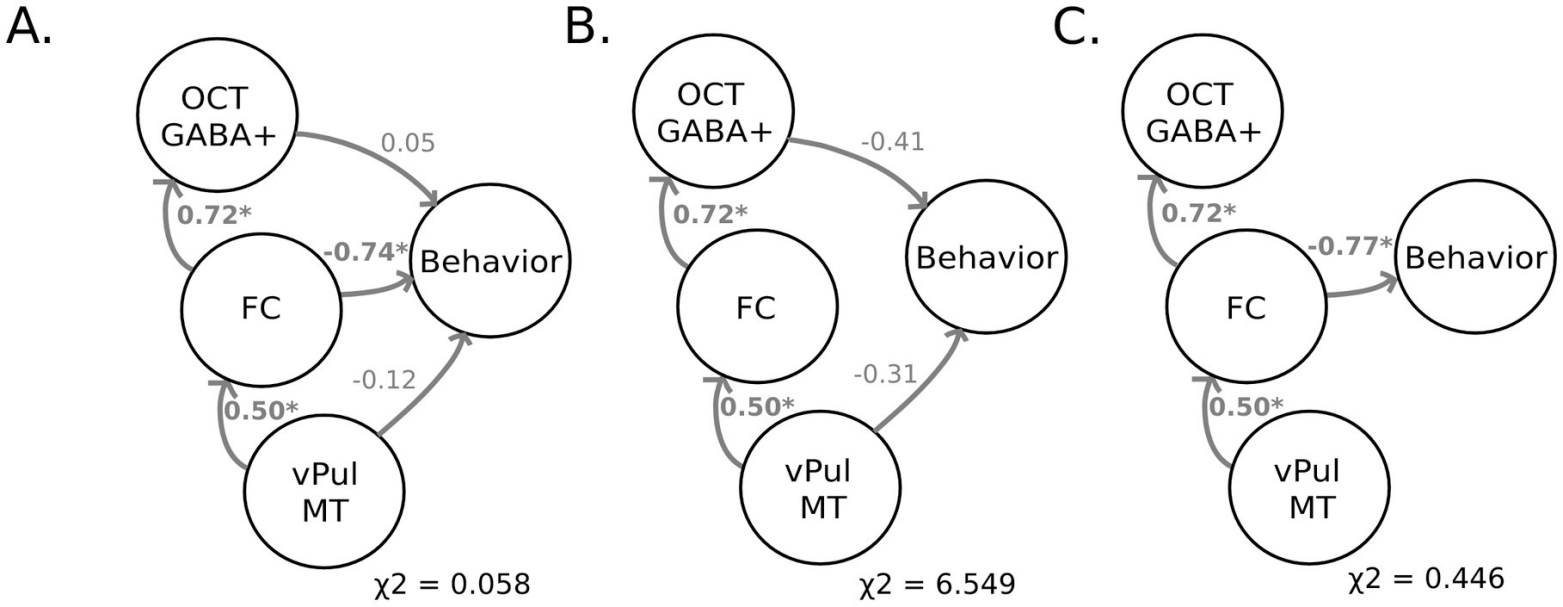
Linking microstructural, functional, and GABAergic plasticity to learning. **(A)** SEM modeling (χ^2^ = 0.058, *p* = 0.810) showed thalamocortical connectivity was a key predictor of behavioral improvement. (**B**) Setting this path to zero resulted in a significantly poorer fit to the data (χ^2^ difference = 6.491, *p* = 0.011). (**C**) Setting to zero the path of OCT GABA+ to behavior or pulvinar MT to behavior resulted in a similar model fit (χ^2^ difference = 0.388, *p* = 0.823) that did not differ significantly from the main model fit. Path lines and coefficients (completely standardized solution, β_STD_) are shown in grey (*n* = 14). Source data are provided at: https://doi.org/10.17863/CAM.93457. MT, magnetization transfer; OCT, occipito-temporal cortex.

We then employed structural equation modeling to test the role of this multimodal plasticity in learning for perceptual decisions. We demonstrate a key role of thalamocortical connectivity in linking thalamic myelin with visual GABAergic plasticity and predicting behavioral improvement. In particular, we tested a model with the following paths: (a) learning-dependent changes in pulvinar MT predict changes in thalamocortical connectivity; (b) learning-dependent changes in thalamocortical connectivity predict changes in OCT GABA+; and (c) learning-dependent changes in pulvinar MT, thalamocortical connectivity, and OCT GABA+ predict changes in behavior. Path directionality in our model—noting that structural equation models with reversed directionality are equivalent when bivariate relationships are modeled—was guided by (a) previous work on the role of pulvinar in regulating interactions between attentional and visual networks [40,41] and (b) our mediation analysis showing that functional connectivity mediated the effect of pulvinar myelination on OCT GABA+. This model (Fig 5A) showed a good fit to the data (df = 1.0, χ^2^ = 0.058, *p* = 0.810, SRMR = 0.012) and the following significant interactions: (a) changes in pulvinar MT predicted changes in thalamocortical connectivity (β_STD_ = 0.504, *p* = 0.029); (b) changes in thalamocortical connectivity predicted changes in OCT GABA+ (β_STD_ = 0.72, *p* < 0.001); and (c) changes in thalamocortical connectivity predicted changes in behavior (β_STD_ = −0.74, *p* = 0.005). Changes in OCT GABA+ (β_STD_ = 0.05, *p* = 0.831) and pulvinar MT (β_STD_ = −0.12, *p* = 0.546) did not predict significantly changes in behavior when accounting for thalamocortical connectivity. To further interrogate the role of thalamocortical connectivity as a key predictor of behavior, we constrained the path between thalamocortical connectivity and behavior to zero; this model resulted in a poor fit (Fig 5B; df = 2.0, χ^2^ = 6.549, *p* = 0.038, SRMR = 0.101 χ^2^ difference = 6.491, *p* = 0.011). In contrast, constraining to zero the paths of OCT GABA+ to behavior and pulvinar MT to behavior did not significantly affect the model fit (Fig 5C; df = 3.0, χ^2^ = 0.446, *p* = 0.931, SRMR = 0.031, χ^2^ difference = 0.388, *p* = 0.823). These results suggest a key role of thalamocortical interactions in predicting behavioral improvement due to training. Further, a model that tested the link between visual-hippocampal connectivity, hippocampal MT, and OCT GABAergic plasticity resulted in a poor fit (i.e., df = 1, χ^2^ = 10.231, *p* = 0.001, SRMR = 0.219), suggesting that our results are specific to thalamocortical connectivity.

Taken together, modeling interactions between MRI measures of myelin, GABA, and functional connectivity indicates a tight interplay between microstructural and functional plasticity mechanisms for perceptual decisions. Our findings propose that adaptive myelin plasticity in pulvinar links to GABAergic visual processing through thalamocortical connectivity to support improved perceptual decisions.

## Discussion

Here, we provide evidence that structural myelin plasticity in subcortical regions interacts with functional mechanisms of cortical neurotransmission to support learning. In contrast to previous studies that have focused on cortical circuits (i.e., frontoparietal and sensory areas) of learning [42] and decision-making [43], we provide evidence that thalamocortical plasticity plays a key role in improving perceptual decisions through training. Using a multimodal quantitative imaging approach, we demonstrate that adaptive myelination supports perceptual decision-making through thalamocortical interactions with neurochemical plasticity mechanisms in visual cortex. Our findings advance our understanding of experience-dependent plasticity mechanisms that support perceptual decision-making in the following main respects.

First, we demonstrate that training increases thalamic myelination, as indicated by learning-dependent changes in grey matter MT measured by quantitative MRI (MPM). Despite the fact that MT imaging measures myelin indirectly by imaging water protons within and close to the myelin sheath, recent work shows that MT maps have high reliability and are strongly linked to histological measures of myelin content [16–18]. Further, quantitative MRI (MPM) allows us to measure not only white but also grey matter myelin and test directly the link between microstructure and neural processing in grey matter. While white matter myelination has been associated with increased transmission speed along long-range axons, grey matter myelination has been associated with microcircuit function related to learning [44] and may serve to improve local activity synchronization [4]. Previous human brain imaging studies, using standard structural MRI or diffusion tensor imaging, have focused on learning-dependent changes in white matter in a range of tasks, including motor [45,46] and perceptual [47,48] learning. In contrast, testing learning-dependent changes in grey matter MT, we provide evidence for a tight link between microstructural plasticity and functional neurotransmission for optimized processing within thalamocortical circuits.

Our results support an active role of myelin in adult learning [1,4,5,49], consistent with animal studies showing increased levels of oligodendrocyte precursor cells (OPCs) that promote axon myelination due to training [44,50,51]. Understanding the link between myelination processes and learning (i.e., behavioral improvement) depends on myelination dynamics. Insights in understanding this link come from animals studies showing rapid OPC proliferation and differentiation at initiation of learning or neuronal stimulation in vivo [2,4,44,52]. Further, previous work [46] suggests that prolonged early learning results in higher myelination but reduced behavioral improvement. Consistent with this work, we found a negative relationship between myelin plasticity and behavioral improvement. Additional analyses (S1 Fig) indicate that individuals who found the task more difficult (i.e., showed slower learning rate and weaker improvement) remained at the early phase of learning for longer, resulting in increased myelination. Future studies are needed to determine the precise kinetics of different myelination processes (i.e., OPC proliferation, myelin remodeling) and how they map onto learning across different time scales [6]. More frequent sampling of myelin content during training could capture myelin increase dynamics at the early phase of training.

Second, our results demonstrate that training reorganizes functional networks that are involved in perceptual decision-making during the course of learning and predict behavioral improvement. In particular, thalamocortical connectivity was stronger before training, while functional connectivity between hippocampal and early visual cortex regions was stronger after training. These results suggest that thalamocortical networks support performance at early stages of learning (initial exposure and engagement with the task), when task demands are higher. This is consistent with the role of pulvinar in regulating visual processing and the role of thalamocortical networks in coordinating visual attentional processing [21,41,53,54]. In contrast, refining feature representations for target identification involves hippocampal and early visual regions that engage later in training. These results are consistent with previous studies implicating the HC in improved task performance following training [55,56]. Further, the involvement of early compared to higher visual areas at later stages of learning [57] has been suggested to afford finer processing of visual information at higher spatial resolution for detecting targets in clutter [58]. This is supported by previous perceptual learning studies showing learning-induced changes in synaptic strength that are associated with LTP-like processes in early visual cortex [59].

Third, we provide evidence that thalamocortical connectivity links thalamic myelin plasticity to visual GABAergic plasticity. In particular, we show that decreased visual cortex GABA—as measured by MRS—due to multisession training in the SN task relates to behavioral improvement, extending our previous work measuring GABAergic plasticity within a single training session [15,23]. Reductions in cortical GABA have been shown to increase neuronal gain by reducing shunting inhibition through tonic GABA receptors [60]. Despite our currently limited knowledge on the neural origins of MRS-GABA [61], it is possible that GABAergic inhibition in visual cortex serves as a gain control mechanism that supports our ability to detect task-relevant features for target identification, while filtering out background noise [15,23].

Importantly, modeling thalamocortical connectivity, pulvinar MT, and visual GABA signals suggests that myelin plasticity in the pulvinar supports learning through thalamocortical interactions with GABAergic inhibition in visual cortex. It is likely that adaptive myelination in pulvinar results in learning-dependent changes in thalamocortical connectivity and GABAergic plasticity in visual cortex to support optimized perceptual decisions, consistent with the role of pulvinar in regulating visual processing [40,41]. In particular, connections from pulvinar to visual cortex have been suggested to regulate inhibitory processing by synapsing directly onto GABAergic interneurons, promoting oscillatory activity and thalamocortical connectivity [41]. Here, we provide evidence that these interactive plasticity mechanisms are key in supporting not only sensory processing but also learning of complex cognitive skills (i.e., identifying targets in cluttered scenes).

Finally, our study sample was limited to male participants due to potential confounding effects of menstrual cycle on GABA measurements. This has been the topic of extensive research [62–67] with several studies restricting MRS-GABA studies to males (e.g., [68–76]). Key hormones (estrogen, progesterone) exerting a suppressive or facilitatory effect on GABA transmission [77, 63–65] may confound within-subject GABA measurements over time. Developing precise methods for controlling for the effects of menstrual cycle on MRS GABA measurements is hampered by physiological complexity (i.e., phase and regional effects of menstrual cycle on GABA) and limited knowledge of the kinetics of menstrual cycle GABA changes in humans. As our study involves repeated MRS GABA measurements over time, it is not possible to satisfactorily control for menstrual cycle effects; i.e., not only the phase and duration of the menstrual cycle but also the kinetics of GABA changes across menstrual cycle days would likely differ substantially across participants. Ongoing large-scale multisite studies are expected to provide normative data that will advance our understanding of these cyclical effects, allow us to develop precise control methodologies, and conduct similar studies including female participants to enhance the generalizability of our findings.

In sum, our findings provide evidence for a tight interplay between microstructural plasticity and functional neurotransmission mechanisms in subcortico-cortical networks for perceptual decision-making. We propose that myelin plasticity in the pulvinar early in the training may regulate—through thalamocortical connectivity—GABAergic gain control mechanisms in visual cortex for selecting task-relevant features. In contrast, as performance becomes more automated later in the training, connectivity across hippocampal and early visual networks increases to facilitate finer processing of task-relevant features and support the identification of targets in clutter. Capturing the dynamics of microstructural (i.e., myelin) and functional (i.e., neurochemical) interactions that drive experience-dependent plasticity is key for understanding how experience molds the adult brain and supports our ability for adaptive behavior across the lifespan.

## Materials and methods

### Participants

Twenty-two males (mean age: 23.45 ± 4.21 years) participated in the study. We did not recruit females to avoid previously reported menstrual cycle effects on GABA concentration [63]. All participants were right-handed, had normal or corrected-to-normal vision, were not under any prescription medication, and gave written informed consent. Participants were naive to the aim of the study and received payment for their participation. All experiments were approved by University of Cambridge Ethics Committee [PRE.2017.057].

### Stimuli

Participants were trained to distinguish radial versus concentric Glass patterns [78] embedded in noise (SN task). Stimuli (size = 7.9° × 7.9°) comprised of white dot pairs (dipoles) that were presented within a square aperture on a black background. The stimulus parameters followed previous studies [15,23]. The dot density was 3%, and the Glass shift (i.e., the distance between 2 dots in a dipole) was 16.2 arc min. The size of each dot was 2.3 × 2.3 arc min^2^. Radial and concentric patterns were generated by placing dipoles orthogonally (radial) or tangentially (concentric) to the circumference of a circle centered on the fixation dot. The spiral angle was defined as the angle between the dot dipole orientation and the radius from the dipole center to the center of the stimulus aperture. For radial patterns the spiral angle was 0° and for concentric 90°. Each stimulus consisted of dot dipoles aligned according to either the radial or concentric spiral angle and noise dipoles for which the spiral angle was randomly selected. The ratio of signal to noise dipoles defined the stimulus signal level; stimuli were presented at 24% ± 1% signal level; i.e., 76% of the dipoles were presented at random position and orientation based on [79].

We randomized the presentation of clockwise (0° to 90° spiral angle) and counterclockwise patterns (0° to −90° spiral angle) across participants. To control for potential local adaptation and ensure that learning related to global shape rather than local stimulus features, we generated a new pattern per trial and jittered (±1 to 3°) the spiral angle across stimuli. Stimuli were presented at the left hemi-field (11.6 arc min from fixation) contralateral to the MRS voxel position to maximize stimulus-related MRS signals, similar to our previous studies [23].

### Experimental design and procedures

Participants took part in 6 experimental sessions over 9 days: 3 brain-imaging sessions (day 1, day 5, and day 9) and 3 consecutive behavioral training sessions (day 6, day 7, and day 8).

#### MRI sessions

All brain imaging sessions followed the same procedure and were conducted with scanner and room lights off. First, T1w, PDw, and MTw FLASH MPM images were acquired, during which participants watched a neutral nature documentary (Dynasties; BBC) with episodes randomized across sessions for each participant. Next, MRS was acquired from OCT and PPC voxels (order counterbalanced across participants). During MRS acquisitions, participants performed the SN task. Participants were asked to judge if a presented stimulus was radial or concentric, responding by button press with the right index finger. For each trial, the stimulus was presented for 300 ms and was followed by variable fixation for 500 to 2,500 ms (a blank screen with a central fixation dot) during which participants were instructed to respond. Participants were not given feedback in the MRI sessions. Participants completed blocks of 200 self-paced trials each (i.e., the next trial was initiated following the participant’s response) comprising 100 trials of concentric and 100 trials of radial patterns. The average number (±SD) of trials completed per scan across participants was as follows: (a) baseline scan: 593.52 ± 135.37; (b) pre-training scan: 645.58 ± 81.78; and (c) post-training scan: 686.21 ± 60.18. Following the MRS acquisition, rs-fMRI was collected while participants fixated on a white central cross on a black background.

#### Training sessions

Behavioral training sessions were conducted in the lab. For each session, participants were trained for 8 runs of 200 trials each, receiving in total 800 trials of concentric and 800 trials of radial patterns. For each trial, participants were presented with a radial or concentric pattern for 300 ms. Participants were asked to judge whether the stimulus presented was radial or concentric. Trial-by-trial feedback was provided by means of a visual cue (green tick for correct, red “x” for incorrect), which remained on the screen for 200 ms and was followed by a fixation dot for a variable time between 500 and 1,500 ms before the next trial onset.

### Data acquisition

#### MRI data acquisition

MRI scans were conducted at the Wolfson Brain Imaging Centre, Cambridge, UK, on a Siemens 3T Prisma (Siemens, Erlangen) with a 32-channel head coil. Whole-brain MPM data were collected using a spoiled multi-echo 3D fast low-angle shot (FLASH) protocol [80] of 3 gradient acquisitions: MT saturation, T1-weighted, and proton density (PD) weighted maps. All weighted maps had 0.8 mm isotropic resolution, field of view of 256 × 240 × 176 mm, and readout bandwidth of 488 Hz/pixel and were collected with partially parallel imaging in each phase-encoded (AP, RL) direction (GRAPPA, 40 integrated autocalibrating lines in each direction, acceleration factor of 2). We used a semiquantitative MT saturation (MTsat) sequence that accounts for spatially varying T1 and B1+, enhancing specificity to myelin content [81,82]. For MT (excitation flip angle of 6°), we acquired 6 gradient echoes with alternating readout gradient polarity at echo times ranging from 2.30 to 18.40 ms in steps of 2.30 ms. For PD (excitation flip angle of 6°) and T1 (excitation flip angle of 21°), we acquired 8 gradient echoes with alternating readout gradient polarity at echo times ranging from 2.30 to 18.40 ms in steps of 2.30 ms. Unaccelerated 8 mm isotropic head and body coil sensitivity bias fields (TR: 6 ms, TE 2.20 ms, flip angle: 6°) were collected before each FLASH acquisition. To correct for field inhomogeneities and susceptibility distortions, we collected B1 and B0 fieldmaps. B1 field maps were acquired with 11 spin-echo and stimulated spin-echo pairs (TR: 500 ms, 4 mm isotropic resolution, 3D-EPI readout with 0.5 ms echo spacing, echo time 39.06 ms, mixing-time 33.8 m) with flip angle between 115° to 65° in 5° increments ([83] for full description of the 3D-EPI B1+ mapping). B0 maps were acquired with a flip angle of 60° (TR: 1020 ms, 3 × 3 × 2 mm resolution. Two images with echo times of 10.00 and 12.46 ms at 260 Hz/pixel bandwidth were acquired, and the phase difference image was generated by Siemens software.

#### MRS data acquisition

MRS spectra were acquired using a MEGA-PRESS sequence [84] (S4 Table) with TR = 3,000 ms; TE = 68 ms; 256 transients (2,048 samples) in 13-minute experiment time. A 14.28-ms Gaussian editing pulse applied at 1.9 ppm (MEGA-ON) and 7.5 ppm (MEGA-OFF). Measurements with this sequence at 3T have been previously shown to produce reliable and reproducible estimates of GABA+ [85]. Water suppression was achieved using variable power with optimized relaxation delays and outer volume suppression. MRS voxel B0 shimming was conducted with automated shimming; the full width at half-maximum measured of the unsuppressed water signal was above 20 Hz after 3 attempts at automated phase mapping, manual shimming was performed. A total of 16 unsuppressed water spectra were collected for eddy current correction and metabolite referencing.

Spectra were acquired from the right OCT and PPC, voxel size 2 × 2 × 2.5 cm. To ensure consistent voxel placement across training sessions and between participants (Fig 4A and S2C Fig), the MRS voxel was manually positioned on each participant’s T1w FLASH anatomical image using anatomical landmarks (superior temporal gyrus and middle occipital gyrus for OCT and intraparietal sulcus for PPC). Voxel position was similar across sessions (mean difference in position between voxel center (mm): OCT pre-training–baseline, X; *M* = 0.62, *SD* = 0.70, Y; *M* = 1.07, *SD* = 0.87, Z; *M* = 1.29, *SD* = 1.03, post-training–pre-training, X; *M* = 0.44., *SD* = 0.49, Y; *M* = 0.76, *SD* = 0.84, Z; *M* = 1.20, *SD* = 1.17. PPC pre-training–baseline, X; *M* = 0.93, *SD* = 0.63, Y; *M* = 0.98, *SD* = 1.05, Z; *M* = 0.93, *SD* = 0.74, post-training–pre-training, X; *M* = 0.49, *SD* = 0.73, Y: *M* = 0.84, *SD* = 0.64, Z: *M* = 1.02, *SD* = 0.81. The mean GM tissue fraction for baseline, pre-training, and post-training was 56.02%, 56.28%, and 56.51% for OCT and 46.62%, 46.75%, and 45.70% for PPC. GM tissue content did not differ significantly between sessions, OCT F_2,34_ = 0.196, *p* = 0.822; PPC F_2,34_ = 1.280, *p* = 0.292.

#### Resting-state fMRI (rs-fMRI)

Echo-planar imaging (EPI) acquisitions with full brain coverage (TR: 727 ms, TE: 34.6 ms, slices: 72; voxel size: 2 mm isotropic; multiband factor: 8; flip angle 48°; volumes: 812) were collected. Cardiac and respiratory signals were recorded using a pulse oximeter and respiratory belt, respectively.

### Data analysis

#### Behavior

We calculated performance accuracy (i.e., percentage correct responses) per session, as: (total correct responses / total trials) * 100. To quantify behavioral improvement, we calculate the difference in post-training and pre-training percent correct scores (post-training–pre-training). When controlling for the baseline session, the percent correct score at baseline was regressed from the post-training–pre-training improvement. Data were excluded from 2 participants who did not show improvement in the behavioral task, as defined by a positive learning rate across sessions (pre-training to post-training).

#### MPMs

We used the hMRI toolbox [82] in in SPM12 (v7771; Wellcome Centre for Human Neuroimaging, London, UK) for MPM map generation and pre-processing. This quantitative MRI and transmit field mapping sequence results in quantitative MPM with improved sensitivity and reliability [81,86]. First, baseline, pre and post T1w images (first echo) were segmented in SPM and brain-masked prior to longitudinal registration with the CAT12 toolbox (http://www.neuro.uni-jena.de/cat/). Brain-masked T1w images from each session were coregistered to the subject-average T1w, and the transformation applied to all MPM scans acquired in that session. The hMRI auto-reorient module was used to reorient images to the anterior commissure, using the first session T1 as a reference for each subject. The hMRI toolbox map creation module was used to generate bias-corrected R1, R2, MT, and PD maps. Five participants were excluded from MPM analyses due to poor map quality as assessed by a PD map error estimate less than 8% (mean / SD white matter intensity) (mean ± SEM: baseline: 6.32% ± 0.12, pre-training: 6.36% ± 0.17, post-training, 6.29% ± 0.17) and visual inspection. One of these participant was also a bivariate outlier (as identified using the Robust Correlation Toolbox [87]) for all MRI and MRS measurement correlations with behavior and was excluded from all further analyses. Clusters from whole-brain MT analysis were masked with the brainnetome [88] and probabilistic thalamus atlases [89] (S2 Table). We concentrated on right hemispheric subregions, i.e., contralateral to stimulus presentation (left hemi-field).

Next, maps were resliced to native space and segmented to create a single tissue-class per subject. Subject maps were normalized to MNI space using DARTEL (“Diffeomorphic Anatomical Registration using Exponentiated Lie algebra”) [90]. Finally, tissue-weighed smoothing [91] was applied with a Gaussian kernel of 6 mm full-width half maximum.

#### MRS

MRS data were pre-processed using MRspa v1.5c (https://www.cmrr.umn.edu/downloads/mrspa/). Spectra were eddy current corrected (ECC2 + zero phase), frequency aligned (3.01 ppm), and phase corrected (least squares) before averaged MEGA-OFF and MEGA-ON spectra were subtracted. LC-model [92] was used to estimate metabolite concentrations by fitting modeled γ-amino-butyric acid (GABA), Glutamate (Glu), Glutamine (Gln), and N acetylaspartate (NAA) to the edited spectra. We refer to GABA concentration as GABA+, as MRS measurements of GABA with MEGA-PRESS include coedited macromolecules [93]. To ensure results were not driven by the chosen reference, GABA+ and Glu concentrations were referenced to both Water and NAA [94].

All spectral linewidths were below 10 Hz and GABA+ Cramer–Rao Lower Bound (CRLB) values were less than 10% with no visible lipid contamination (as detected by visual inspection by 2 independent reviewers (PF, JZ). Signal-to-noise ratio (SNR) was calculated as the amplitude of the NAA peak in the difference-spectrum divided by twice the root mean square of the residual signal [92]. We have not included control analyses for changes in CRLB, as reductions in GABA concentration have been shown to be inherently linked to increases in CRLB [94–96]. Two participants were excluded from MRS analyses (one had inconsistent voxel placement across sessions (only 25% overlap), one was detected as univariate outlier (post-training–pre-training) using MATLAB isoutlier function (Grubbs test) [97]. The voxel percentage of grey matter (GM), white matter (WM), and cerebrospinal fluid (CSF) in the OCT and PPC MRS voxels was calculated from the segmented MPM maps. Correction for voxel tissue composition was performed by (1) dividing GABA+ concentration by 1 –fCSF [98]; (2) regressing CSF voxel fraction from measured GABA+; and (3) accounting for GM voxel fraction with the α-correction method [99]. OCT mask used in all analysis was the 50% overlap of MRS voxels across participants (Fig 4A and S2 Table).

#### Resting-state fMRI (rs-fMRI)

rs-fMRI data were pre-processed in SPM12 following the HCP pipeline for multiband data [100]. Two dummy scans were removed; EPI data were distortion corrected (Fieldmap toolbox) [101] and motion corrected. One participant was excluded from resting-state analysis due to high head movement (>2 mm). Data were not slice-time corrected [100]. For EPI coregistration, the high-resolution single-band reference was used for alignment estimation. Subject EPI images were aligned across sessions using CAT12 longitudinal alignment module. Subject R1 maps (coregistered across sessions; see MPM pre-processing) were averaged, segmented, and brainmasked. Subject EPIs were aligned to the average R1 anatomical and normalized with DARTEL deformation fields generated during MPM pre-processing. Data were resliced after MNI normalization to minimize the number of interpolation steps. After normalization, data were skull stripped, smoothed (4 mm Gaussian kernel), wavelet despiked with the BrainWavelet toolbox [102], and linear drifts removed (linear detrending).

Data were denoised using spatial group independent component analysis (ICA; GIFT Toolbox v3.0b; http://mialab.mrn.org/software/gift/). Principal component analysis was applied for dimensionality reduction, first at the subject level, then at the group level. A total of 35 components were extracted from the data, specified manually to yield components with clear visually identifiable components. Group information guided ICA (GIG-ICA) back-reconstruction was used to reconstruct subject-specific components from the group components. We visually inspected the results and identified noise components according to published procedures [103]. Using consensus voting among 2 experts (JZ, VK), we labeled 11 of the 35 components as noise that captured signal from veins, arteries, CSF pulsation, susceptibility, and multiband artifacts. A soft cleanup approach [104] was used to clean the data. Motion parameters (translation, rotation, and their squares and derivatives) were regressed from each voxel and ICA component time course. The unique contribution of each ICA component to the voxel time-course was estimated with multiple regression and subtracted.

Physiological regressors were generated from heart rate (third order), respiratory recordings (fourth order), and their interaction (first order) with zero delay for HRV and RVT terms using the PhysIO toolbox [105]. Data were treated for serial correlations using the FAST autoregressive model [106] with physiological and motion (translation, rotation, and their squares and derivatives) regressed from the timeseries. The first eigenvariate were extracted from regions of interest (ROIs) and fifth order Butterworth band-pass filtered (0.01 to 0.08 Hz). Early visual brain regions (V1, V2, V3, V4) were identified based on a probabilistic atlas [107]. ACC was defined by whole-brain functional connectivity analysis seeded in the pulvinar (thresholded *p* < 0.005) that yielded a cluster in dorsal ACC. We used the MNI coordinates for this cluster (x = 4, y = 20, z = 38) as the center of a spherical mask (radius 8 mm^3^) that defined the ACC ROI for further analysis. We computed the first eigenvariate across all GM voxels within ROIs to derive a single time course per ROI. Functional connectivity was computed as the Pearson correlation between the first eigenvariate of 2 ROIs and the variance was normalized with fisher-z transform before mean-change analyses.

Network connectivity was calculated as the average strictly (i.e., not including the diagonal) upper-triangular of the node-to-node correlation matrix (thalamocortical: OCT, pulvinar, ACC; visual-hippocampal: V1, V2, V3, V4, HC).

### Statistical analysis

Repeated measure ANOVAs were used to assess change in behavioral improvement across all experimental sessions and network connectivity across scanning sessions with JASP v0.14.1. MT change across scanning sessions was analyzed with a mass-univariate repeat-measures GLM (contrast [–1, –1, 2]) in SPM12 (*p* < 0.05 cluster-wise FWE correction with a cluster defining threshold of *p* < 0.001). Violations of sphericity detected by Mauchley’s test were corrected with the Greenhouse–Geisser method.

For correlation analyses, difference between pre-training and post-training sessions were calculated for all measures. To control for baseline variability, baseline session values were regressed from post-training to pre-training differences. Confirmatory analyses were undertaken by generating bootstrapped confidence intervals (600 resamples) of the correlation coefficient; significance is determined when the CI does not include zero. Correlations were performed with the Pearson function of the Robust Correlation Toolbox [87]. Correlations were compared using Steigers Z. Mediation analysis and structural equation modeling was conducted with JASP v0.14.1. Model fit was assessed using the chi-squared (χ^2^) test and standardized root mean square of the residuals (SRMR), on the difference between the empirical covariance matrix and model-implied covariance matrix.

## Supporting information

S1 Fig Correlating subcortical MT with behavioral improvement. The correlation between behavioral improvement and MT change (post-training minus pre-training) in (**A)** vPul (r = −0.49, *p* = 0.045, CI [−0.82, −0.01]) and (**B)** HC (r = −0.59, *p* = 0.012, CI [−0.90, −0.05]) remained significant when accounting for baseline variability ((**C**) vPul: r = −0.58, *p* = 0.015, CI [−0.80, −0.22]; (**D)** HC: r = −0.62, *p* = 0.008, CI [−0.89, −0.11]) (i.e., regressing out baseline measures). Superior colliculus MT change was significantly correlated with behavioral improvement (r = −0.53, *p* = 0.028, CI [−0.83, −0.07]; baseline regression (r = −0.58, *p* = 0.015, CI [−0.88, −0.19]). Further analysis on the superior colliculus was not performed due to the small number of voxels in this region (S2 Table). There were no significant correlations between changes in MT and behavior (performance accuracy) before training (pre-training minus baseline) (vPul: r = 0.0002, *p* = 0.999, CI [−0.59, 0.53]; HC: r = −0.02, *p* = 0.929, CI [−0.53, 0.41]). Source data are provided at: https://doi.org/10.17863/CAM.93457. Considering individual variability in learning rate provides some insight into the negative correlation between MT changes and behavioral improvement. In particular, individuals who improved the most after training learned faster, as indicated by a significant correlation between learning rate and behavioral improvement (r = 0.75, *p* < 0.001, CI [0.42, 0.90]). Further faster learners showed lower MT change after training, as indicated by a significant correlation (r = −0.592, *p* = 0.010, [−0.82, −0.20) between learning rate (calculated across 6 sessions from pre- to post-training) and MT change (post-training minus baseline). Interestingly, faster learners showed significantly (t_15_ = 3.37, *p* = 0.004) lower MT change (*n* = 9; mean learning rate = 3.84; mean behavioral improvement = 11.13%; mean MT change = 1.21%) compared to slower learners who showed higher MT change (*n* = 9; mean learning rate = 1.05; mean behavioral improvement = 3.89%; mean MT change = 9.67%). These analyses suggest that learning-dependent myelin plasticity is stronger when participants find the task difficult and learn slower, consistent with previous work showing that prolonged early learning promotes myelination [46]. HC, hippocampus; MT, magnetization transfer; vPul, ventral pulvinar.
(TIFF)

S2 Fig Learning-dependent changes in white matter MT. **(A)** A repeated-measures whole brain GLM on white matter MT, using the same parameters as the analysis on grey matter showed a significant white matter cluster (binary mask) adjacent to the Th–HC cluster. (**B**) Significant negative correlation between change in MT in the white matter cluster adjacent to the Th–HC cluster and behavioral improvement (r = −0.59, *p* = 0.013, CI [−0.81, −0.19]). This remained significant after baseline regression (r = −0.61, *p* = 0.010, CI [−0.82, −0.29]). There was no significant correlation between change in white matter MT and behavior before training (pre-training minus baseline) (r = 0.011, *p* = 0.967, CI [−0.52, 0.62]). Source data are provided at: https://doi.org/10.17863/CAM.93457. MT, magnetization transfer; Th–HC, thalamic-hippocampal.
(TIFF)

S3 Fig Controls for correlations with OCT GABA+. **(A)** Correlations of OCT GABA+ change (post-training minus pre-training) with behavioral improvement remained significant after accounting for baseline variability (i.e., regressing out baseline measures) (r = −0.65, *p* = 0.005, CI [−0.89, −0.25]). There was no significant correlation between OCT GABA+ change and behavior before training (pre-training minus baseline) (r = −0.03, *p* = 0.917, CI [−0.47, 0.39]). (**B)** Example OCT representative MEGA-PRESS spectra showing measured difference spectra, spectral fit, residual and GABA fit (e.g., GABA multiplet at 3 ppm). There were no significant differences in data quality measures across sessions for these voxels (one-way repeat measures ANOVA): Linewidth (OCT: F_2,32_ = 2.06, *p* = 0.143, PPC: F_2,32_ = 0.14, *p* = 0.867), SNR (OCT: F_2,32_ = 0.05, *p* = 0.945, PPC: F_2,32_ = 0.22, *p* = 0.80), CRLB (OCT: F_2,32_ = 2.01, *p* = 0.15, PPC: F_2,32_ = 0.56, *p* = 0.579). (**C)** Group MRS voxel mask (cortical region common in 50% or more of participants) indicating the position of the PPC (control) voxel. (**D)** PPC GABA+ change was not significantly correlated (r = 0.36, *p* = 0.154, [−0.22, 0.72]) with behavioral improvement (*shown*; following regression of baseline measures; r = 0.34, *p* = 0.186, [−0.15, 0.76]). Source data are provided at: https://doi.org/10.17863/CAM.93457. CRLB, Cramer–Rao Lower Bound; MRS, magnetic resonance spectroscopy; OCT, occipito-temporal cortex; PPC, posterior parietal cortex; SNR, signal-to-noise ratio.
(TIFF)

S1 Table Significant clusters from whole-brain GLM on grey matter MT (one-way repeated measures ANOVA: baseline: -1, pre-training: -1, post-training: 2). For each cluster, the number of voxels, x, y, z coordinates of the peak voxel and cluster-level significance are shown.
(DOCX)

S2 Table Masks for MT (voxel size: 0.8 mm isotropic) and rs-fMRI (voxel size 2 mm isotropic) analyses extracted from the Brainnetome atlas [88] and probabilistic thalamus [89] and visual [107] atlases. Masks contain only grey matter voxels, as determined from the grey matter segmentation of the group average anatomical scan. Number of voxels and MNI coordinates are shown. V2 and V3 masks include dorsal and ventral subregions.
(DOCX)

S3 Table MRS GABA+ quality measures (mean and standard deviation per scanning session). There were no significant differences in data quality measures across sessions (one-way repeated measures ANOVA): Linewidth (OCT: F_2,32_ = 2.06, *p* = 0.143, PPC: F_2,32_ = 0.14, *p* = 0.867), SNR (OCT: F_2,32_ = 0.136, *p* = 0.718, PPC: F_2,32_ = 0.01, *p* = 0.92), CRLB (OCT: F_2,32_ = 2.01, *p* = 0.15, PPC: F_2,32_ = 0.56, *p* = 0.579).
(DOCX)

S4 Table Minimum reporting standards in MRS checklist. (DOCX)

## Abbreviations

ACC: anterior cingulate cortex
CRLB: Cramer–Rao Lower Bound
CSF: cerebrospinal fluid
EPI: echo-planar imaging
FLASH: fast low-angle shot
GABA: γ-amino-butyric acid
HC: hippocampus
IFG: inferior frontal gyrus
ITC: inferior temporal cortex
MPM: multiparameter mapping
MRS: magnetic resonance spectroscopy
MT: magnetization transfer
NAA: N acetylaspartate
OCT: occipito-temporal cortex
OPC: oligodendrocyte precursor cell
PD: proton density
PPC: posterior parietal cortex
ROI: region of interest
rs-fMRI: resting-state fMRI
SN: signal-in-noise
SNR: signal-to-noise ratio
SRMR: standardized root mean square of the residuals
Th–HC: thalamic-hippocampal
vPul: ventral pulvinar
