# Supplementary figures and images for: Microstructural and neurochemical plasticity mechanisms interact to enhance human perceptual decision-making
Source: PLoS Biol. 2023 Mar 10;21(3):e3002029. doi: 10.1371/journal.pbio.3002029 (PMC10032544; doi:10.1371/journal.pbio.3002029)

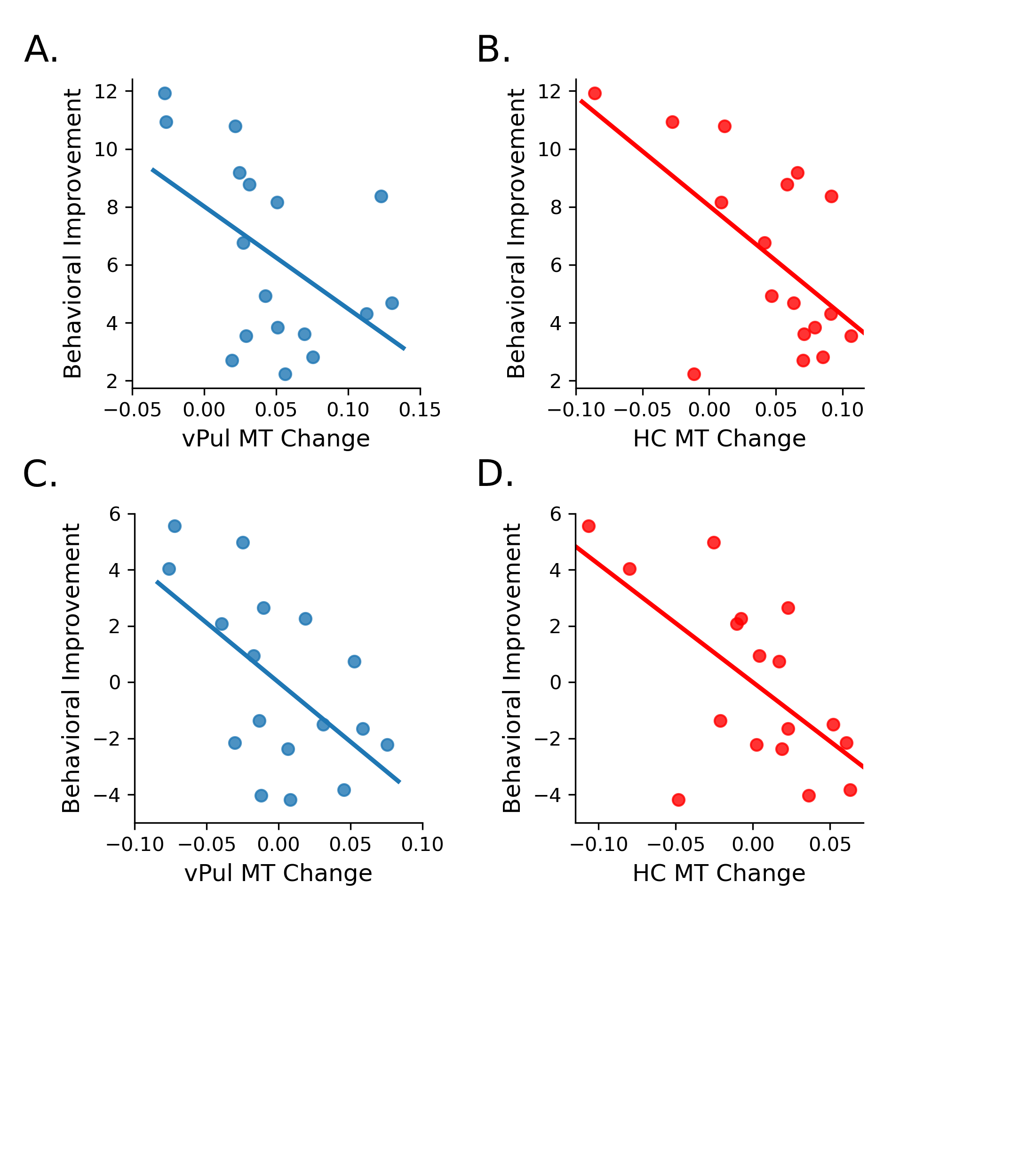

Supplement: S1 Fig — The correlation between behavioral improvement and MT change (post-training minus pre-training) in (A) vPul (r = −0.49, p = 0.045, CI [−0.82, −0.01]) and (B) HC (r = −0.59, p = 0.012, CI [−0.90, −0.05]) remained significant when accounting for baseline variability ((C) vPul: r = −0.58, p = 0.015, CI [−0.80, −0.22]; (D) HC: r = −0.62, p = 0.008, CI [−0.89, −0.11]) (i.e., regressing out baseline measures). Superior colliculus MT change was significantly correlated with behavioral improvement (r = −0.53, p = 0.028, CI [−0.83, −0.07]; baseline regression (r = −0.58, p = 0.015, CI [−0.88, −0.19]). Further analysis on the superior colliculus was not performed due to the small number of voxels in this region (S2 Table). There were no significant correlations between changes in MT and behavior (performance accuracy) before training (pre-training minus baseline) (vPul: r = 0.0002, p = 0.999, CI [−0.59, 0.53]; HC: r = −0.02, p = 0.929, CI [−0.53, 0.41]). Source data are provided at: https://doi.org/10.17863/CAM.93457. Considering individual variability in learning rate provides some insight into the negative correlation between MT changes and behavioral improvement. In particular, individuals who improved the most after training learned faster, as indicated by a significant correlation between learning rate and behavioral improvement (r = 0.75, p < 0.001, CI [0.42, 0.90]). Further faster learners showed lower MT change after training, as indicated by a significant correlation (r = −0.592, p = 0.010, [−0.82, −0.20) between learning rate (calculated across 6 sessions from pre- to post-training) and MT change (post-training minus baseline). Interestingly, faster learners showed significantly (t15 = 3.37, p = 0.004) lower MT change (n = 9; mean learning rate = 3.84; mean behavioral improvement = 11.13%; mean MT change = 1.21%) compared to slower learners who showed higher MT change (n = 9; mean learning rate = 1.05; mean behavioral improvement = 3.89%; mean MT change = 9.67%). Th [file pbio.3002029.s001.tiff]

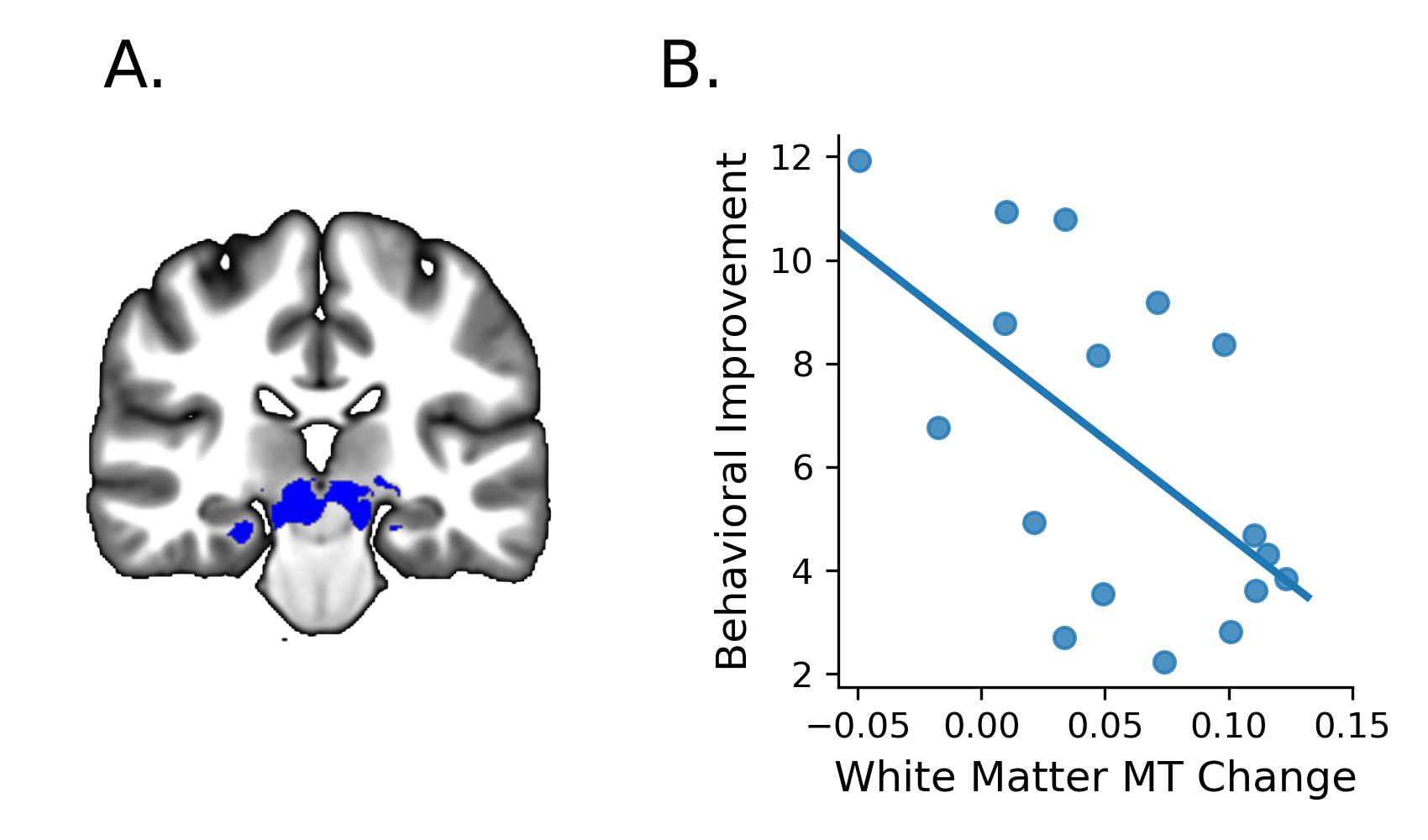

Supplement: S2 Fig — (A) A repeated-measures whole brain GLM on white matter MT, using the same parameters as the analysis on grey matter showed a significant white matter cluster (binary mask) adjacent to the Th–HC cluster. (B) Significant negative correlation between change in MT in the white matter cluster adjacent to the Th–HC cluster and behavioral improvement (r = −0.59, p = 0.013, CI [−0.81, −0.19]). This remained significant after baseline regression (r = −0.61, p = 0.010, CI [−0.82, −0.29]). There was no significant correlation between change in white matter MT and behavior before training (pre-training minus baseline) (r = 0.011, p = 0.967, CI [−0.52, 0.62]). Source data are provided at: https://doi.org/10.17863/CAM.93457. MT, magnetization transfer; Th–HC, thalamic-hippocampal. (TIFF) [file pbio.3002029.s002.tiff]
