# Supplementary material for: Microstructural and neurochemical plasticity mechanisms interact to enhance human perceptual decision-making
Source: PLoS Biol. 2023 Mar 10;21(3):e3002029. doi: 10.1371/journal.pbio.3002029 (PMC10032544; doi:10.1371/journal.pbio.3002029)
Supplement: S1 Table — For each cluster, the number of voxels, x, y, z coordinates of the peak voxel and cluster-level significance are shown. (DOCX) [file pbio.3002029.s004.docx]

| Cluster Location | Hemisphere | Cluster Size | x | y | z | *p* value (Cluster level, FWE corrected) |
| --- | --- | --- | --- | --- | --- | --- |
| Thalamic – Hippocampal | Bilateral | 30997 | 4.8 | -24 | -8.8 | < 0.001 |
| Inferior Frontal Gyrus | R | 939 | 16.8 | 39.2 | -16.8 | 0.015 |
| Inferior Temporal Cortex | R | 1241 | 33.6 | -7.2 | -36.6 | 0.003 |
