# Supplementary material for: Microstructural and neurochemical plasticity mechanisms interact to enhance human perceptual decision-making
Source: PLoS Biol. 2023 Mar 10;21(3):e3002029. doi: 10.1371/journal.pbio.3002029 (PMC10032544; doi:10.1371/journal.pbio.3002029)
Supplement: S2 Table — Masks contain only grey matter voxels, as determined from the grey matter segmentation of the group average anatomical scan. Number of voxels and MNI coordinates are shown. V2 and V3 masks include dorsal and ventral subregions. (DOCX) [file pbio.3002029.s005.docx]

| **Brain Region (right hemisphere)** | Voxel Number (MT, 0.8 mm isotropic voxel) | Voxel Number (rsfMRI, 2 mm isotropic voxel) | X | Y | Z |
| --- | --- | --- | --- | --- | --- |
| Pulvinar | 1279 | 79 | 9.60 | -25.60 | 0.80 |
| Hippocampus | 1710 | 114 | 21.60 | -27.20 | -11.20 |
| Superior Colliculus | 168 | 5 | 5 | -31 | -3 |
| Parahippocampus | 2551 | N/A | 22.40 | -30.40 | -21.60 |
| OCT (GABA+ Voxel) | N/A | 769 | 47.2 | -53.60 | 8.80 |
| ACC | N/A | 180 | 6 | 20 | 38 |
| V1 | N/A | 900 | 8.00 | -80.80 | 6.40 |
| V2d | N/A | 498 | 9.60 | -92.00 | 17.60 |
| V2v | N/A | 687 | 8.80 | -80.00 | -7.20 |
| V3d | N/A | 1772 | 29.60 | -84.00 | 22.40 |
| V3v | N/A | 708 | 17.60 | -68.80 | -9.60 |
| V4 | N/A | 221 | 32.80 | -79.20 | -13.60 |
