# Supplementary material for: Microstructural and neurochemical plasticity mechanisms interact to enhance human perceptual decision-making
Source: PLoS Biol. 2023 Mar 10;21(3):e3002029. doi: 10.1371/journal.pbio.3002029 (PMC10032544; doi:10.1371/journal.pbio.3002029)
Supplement: S3 Table — There were no significant differences in data quality measures across sessions (one-way repeated measures ANOVA): Linewidth (OCT: F2,32 = 2.06, p = 0.143, PPC: F2,32 = 0.14, p = 0.867), SNR (OCT: F2,32 = 0.136, p = 0.718, PPC: F2,32 = 0.01, p = 0.92), CRLB (OCT: F2,32 = 2.01, p = 0.15, PPC: F2,32 = 0.56, p = 0.579). (DOCX) [file pbio.3002029.s006.docx]

| **MRS Measure** | **Voxel** | **Scan** | **Mean** | **Standard Deviation** |
| --- | --- | --- | --- | --- |
| **CRLB** | **OCT** | baseline | 4.65 | 0.49 |
|  |  | pre-training | 4.53 | 0.51 |
|  |  | post-training | 4.82 | 0.40 |
|  | **PPC** | baseline | 4.82 | 0.40 |
|  |  | pre-training | 4.82 | 0.40 |
|  |  | post-training | 4.94 | 0.43 |
| **Linewidth** | **OCT** | baseline | 8.22 | 1.04 |
|  |  | pre-training | 8.18 | 0.56 |
|  |  | post-training | 7.78 | 0.68 |
|  | **PPC** | baseline | 5.89 | 0.98 |
|  |  | pre-training | 5.84 | 0.83 |
|  |  | post-training | 5.95 | 0.57 |
| **SNR** | **OCT** | baseline | 23.12 | 1.27 |
|  |  | pre-training | 23.06 | 1.44 |
|  |  | post-training | 23.00 | 1.37 |
|  | **PPC** | baseline | 26.18 | 1.59 |
|  |  | pre-training | 25.88 | 1.36 |
|  |  | post-training | 26.12 | 2.23 |
