# Supplementary material for: Microstructural and neurochemical plasticity mechanisms interact to enhance human perceptual decision-making
Source: PLoS Biol. 2023 Mar 10;21(3):e3002029. doi: 10.1371/journal.pbio.3002029 (PMC10032544; doi:10.1371/journal.pbio.3002029)
Supplement: S4 Table — (DOCX) [file pbio.3002029.s007.docx]

| Site (Name or Number) | MRC Cognition and Brain Sciences Unit (University of Cambridge) |
| --- | --- |
| 1. Hardware |  |
| a. Field strength [T] | 3 |
| b. Manufacturer | Siemens |
| c. Model | Prisma |
| d. RF coils | 32-channel receive head coil |
| e. Additional hardware | N/A |
| 2. Acquisition |  |
| a. Pulse sequence | MEGA-PRESS |
| b. Volume of Interest (VOI) locations | Occipital Temporal Cortex (OCT), Posterior Parietal Cortex (PPC) |
| c. Nominal VOI size [cm^3^, mm^3^] | 20x20x25 mm |
| d. Repetition Time (TR), Echo Time (TE) [ms, s] | TR=3000ms, TE=68ms |
| e. Total number of Excitations or acquisitions per spectrum | 256 |
| f. Additional sequence parameters: | Spectral bandwidth: 1200 Hz  Spectral points: 2048 |
| g. Water Suppression Method | Water suppression was achieved using variable power with optimized relaxation delays and outer volume suppression. |
| h. Shimming Method, reference peak, and thresholds for “acceptance of shim” chosen | Automated 3D head shim (GRE-BRAIN) to achieve water peak linewidth below 10 Hz. |
| i. Triggering or motion correction method | N/A |
| 3. Data analysis methods and outputs |  |
| a. Analysis software | MRspa (preprocessing, version v1.5c), LCModel (fitting and quantification) |
| b. Processing steps deviating from quoted reference or product | MRspa pre-processing options selected:  - eddy current corr.: ECC2 + zero phase  - frequency corr.: absolute (3.01)  - phase corr.: least square |
| c. Output measure | Tissue-corrected concentrations relative to water or NAA |
| d. Quantification references and assumptions, fitting model assumptions | We fitted model spectra of γ-amino-butyric acid (GABA), glutamate (Glu), glutamine (Gln) and N acetylaspartate (NAA) to the edited spectra.  The model spectra were generated based on previously reported chemical shifts and coupling constants using the GAMMA/PyGAMMA simulation library of VESPA for carrying out the density matrix formalism. A 20 x 20 spatial matrix was used to simulate the spatial variations inside and outside the nominal PRESS dimensions. Simulations were performed with the same RF pulses and sequence timings as that on the 3T system in use. |
| 4. Data Quality |  |
| a. Reported variables | See Table S3 |
| b. Data exclusion criteria | Water peak linewidth > 10 Hz  CRLB > 10%  Lipid contamination by visual inspection |
| c. Quality measures of postprocessing Model fitting | See Table S3 |
| d. Sample Spectrum | See Figure S2 |
